# Nurses’ and nursing students’ reasons for entering the profession: content analysis of open-ended questions

**DOI:** 10.1186/s12912-023-01307-8

**Published:** 2023-05-05

**Authors:** Lisa McKenna, Ian Ruddy Mambu, Christine L. Sommers, Sonia Reisenhofer, Julie McCaughan

**Affiliations:** 1 La Trobe University, Melbourne, Australia; 2 Universitas Pelita Harapan, Tangerang, Indonesia; 3 Bairnsdale Regional Health Service, Bairnsdale, Australia; 4 Siloam Hospitals, Tangerang, Indonesia

**Keywords:** Altruism, Career, Content analysis, Indonesia, Motivation, Nursing, Recruitment, Workforce

## Abstract

**Background:**

Global nursing shortages require effective recruitment strategies and understanding of individuals’ motivations to enter the profession. These can be complex and bound by numerous factors such as gender and culture. While much research around this has been conducted, little has been undertaken in non-Western cultures where motivations could be different.

**Aim:**

To explore Indonesian nurses’ and nursing students’ motivations for entering the nursing profession.

**Design:**

Online survey with closed and open-ended questions drawn from two different studies. This paper reports findings from one similar open-ended question.

**Methods:**

As part of two larger surveys, nurses from 13 hospitals across one private health care group and nursing students with clinical experienced enrolled in a baccalaureate nursing program in Indonesia were asked the question, *Why do you want to be a nurse?* Responses were translated into English and back-translated into Indonesian prior to being subjected to summative content analysis.

**Results:**

In total, 1351 nurses and 400 students provided responses to the question, representing 98.72% and 99.70% respectively of those completing the survey. Both groups were primarily influenced by desire to serve others and God, personal calling and influence of family members and others. Nurses identified a desire to work in the health field and with the sick, in a noble and caring profession.

**Conclusions:**

Nurses and nursing students were motivated by traditional perspectives on nursing. These should be considered in future recruitment activities. However, more research is needed to understand how these factors influence career choice.

## Background

Nurses have been identified as crucial to global achievement of Sustainable Development Goals (SDGs) and play a strong role in health policy, achievement of health targets [1]. However, global workforce shortages, along with an ageing nursing population [1] and COVID-19 related burnout further impacting retention [2], mean that strategies for recruitment into the nursing profession are paramount to meet community health care needs. While more than sufficient numbers of graduate nurses are being produced in Indonesia, 54.1% of nurses are located in urban areas, mostly in Java Island, while the other 45.9% are in rural areas with reported chronic shortages in some areas of the country, especially among communities in Eastern Indonesia [3]. The need for public education to improve the professional image of nursing has been advocated as one means for promoting recruitment of new nurses into the profession [4].

Understanding why individuals seek a career in nursing is important in facilitating the targeting of recruitment strategies. Some studies have reported on individual motivations to enter nursing courses, largely from western countries. In Italy, Messineo et al. [5] found that first year nursing students entered their courses with high levels of empathy and altruistic and prosocial motivations. However, there is also evidence that this declines over the duration of nursing studies [6]. Crick et al. [7] found that new nursing students in the United Kingdom were motivated to enter the course due to a desire to care for others. In a study of graduate entry nursing students in Australia, McKenna et al. [8] identified that previous exposure to nurses, either personally or family, played an important role in their decisions to enter the course, along with desire to care for others.

In a systematic review of 29 papers, Wu et al. [9] examined motivations of healthcare students influencing career choice, identifying a range of both intrinsic and extrinsic factors. They found that altruism through a desire to help others was strong among nursing students, particularly for those who were sick and in need of care. Job security and social status of nursing were considered important, while financial remuneration was not considered as important as for other health professions. Influence of family was mixed in nursing, with some families seeing nursing as having low pay and status, while having family members in the profession was positively influential.

The introduction of the Indonesian Nursing Act in 2014 saw rapid development in the nurse education system and nursing practice with introduction of curriculum standards and accreditation, national competency examination, and nurse registration across the country [10]. Four-year bachelor or three-year diploma courses can be undertaken to become professional or vocational nurses respectively. Furthermore, there has been recent growth in postgraduate and doctoral programs across the country [11]. Few previous studies could be sourced exploring why individuals are motivated (seek) to enter the nursing profession in Indonesia. In one study, 20 nursing diploma students participated in focus groups exploring their reasons for choosing to study nursing. Findings identified wanting to help family and others, being inspired by nurses, wanting to improve the image of nursing, influence of family and parents, and work opportunities all influenced choices [12]. In another study of 57 students in a bachelor degree nursing program, the majority entered the program because they were interested in the nursing profession or wanted to become nurses [13]. Around a third of students were motivated by their parents to enter the program. Previous studies regarding motivation among existing qualified nurses could not be sourced. Hence, this study sought to explore why Indonesian nurses and nursing students pursued nursing careers.

## Methods

Data were drawn from two concurrent studies involving online questionnaires, the first focusing on Indonesian registered nurses’ training needs [14] and the second, on Indonesian nursing students’ experiences of their clinical learning environment [15]. In each study, participants were asked the same open-ended question: *Why do you want to be a nurse?* The responses to that question comprise the focus of this paper. Prior to commencement of data collection, approvals were obtained from ethics committees at La Trobe University (ID: S17-155) and Universitas Pelita Harapan (No.005/MRIN-EC/ECL/III/2018). In the original studies, inclusion criteria for the nurses were currently working at the private hospital with a 3-year nursing diploma level qualification or above. Inclusion criteria for nursing students were those who had completed a clinical placement in the private hospital, were enrolled as a nursing student in the university, and were 18 years of age or older. There were no specific exclusion criteria.

Links to the online Qualtrics surveys were provided in participant information materials. These were circulated via internal email and WhatsApp groups to a convenience sample of 2093 eligible registered nurses from 13 sites of an Indonesian private health care group across Jakarta, Sumatra, Sulawesi and Bali and Nusa Tenggara Timur and 796 students from one nursing degree program. Participants provided informed consent using a survey link in the study information provided and participation was voluntary and anonymous. In total, 406 s- and third-year students and 1355 nurses completed the open-ended question forming the basis of this paper.

Responses to the question from both datasets were translated from Indonesian into English and back-translated by two researchers to ensure original meanings were retained [16]. Data were then subjected to summative content analysis [17]. Key words were initially identified and coded within Microsoft Excel by two members of the research team. Codes were then manually collated into categories of like terms, quantified utilising frequency counts. Overarching themes were then identified from categories.

## Findings

In total, 400 students and 1351 nurses provided responses to the question, representing response rates of 98.52% and 99.70% respectively. Among the student group, the average age was 20 years and 84% were female. In the registered nurse group, 80.8% were female and years of experience were relatively evenly distributed from less than one to more than 10 years. Of these, 39% had been in the profession for longer than five years, that is, before introduction of the Indonesian Nursing Act. From the analysis, seven categories and five themes emerged from the student data (Table 1) and eight categories and four themes from the nurse data (Table 2). Substantial overlap was evident across the two groups and rankings.

**Table 1:** Student data (n = 400)

Ranking	Category	Code count	%	Theme group
1	To help or serve others or own family	200	50.00	Impact others’ lives
2	Personal decision or calling	128	32.00	Decision
3	Influence of family, others or circumstances	89	22.25	Decision
4	Be a part of or improve the profession of nursing	44	11.00	Profession of nursing
5	As a ministry and service to God	38	9.50	Service to God/ religious response
6	Received scholarship offer	34	8.50	Financial
7	To be a blessing to others	17	4.25	Impact others’ lives

**Table 2:** Nurse data (n = 1351)

Ranking	Category	Code count	%	Theme group
1	To help or serve others or own family	607	44.93	Impact others’ lives
2	Personal decision or calling	296	21.91	Decision
3	Desire to work in health field/with the sick	250	18.50	Profession of nursing
4	Caring, noble profession	228	16.88	Profession of nursing
5	Influence of family, others or circumstances	207	15.32	Decision
6	No reason/choice	37	2.74	Decision
7	Received scholarship offer/ financial	28	2.07	Financial
8	Interaction with people	18	1.33	Impact others’ lives

Overwhelmingly, having an *impact on others’ lives* was key to both groups through *helping or serving others or own family* ranked highest for both groups, reported by 50.00% of students and 44.93% of nurses. Students also wanted *to be a blessing to others*, while nurses valued the *interaction with people* that is a fundamental part of nursing practice.

For both groups, factors around *decision* to enter nursing ranked second. Many described having a calling to nursing, while others described this in terms of a ‘childhood dream’, or for ‘personal reward or satisfaction’. *Influence of family, others or circumstances* played an important part for students and nurses, while a number of nurses described having *no reason or choice* around entering nursing. *Service to God/religious response* was also noted to have been the motivation for some students (9.50%); however, it is important to note that these students were enrolled in a faith-based university, so this could be expected. Attraction to the *Profession of nursing* was also identified as an important factor for both groups. Students expressed a desire to be a part of, or improve, the profession, while many nurses identified a desire to work in the health field or with the sick. For nurses, the status of the profession as noble and caring was a strong factor.

Finally, *financial* reasons were identified by a small number of participants in both groups who identified commencing their nursing education as they received an offer of a scholarship.

## Discussion

With a predicted continued global nursing shortage, targeted successful strategies need to be introduced to recruit into the profession. Understanding motivations for entering nursing courses can assist with the development of appropriate recruitment strategies and may also inform future retention strategies to keep nurses within the profession. Hence, this study sought to understand why nurses and nursing students in Indonesia chose to enter the profession. Prior to this study, little was known about such motivations in Indonesia, and outside of western countries.

A desire to impact others’ lives was the strongest reported influence for both nurses and nursing students in this study. This was seen as wanting to serve others, and desire to work with the sick. Altruism has long been identified as a reason why individuals choose nursing careers. However, Carter [18] cautions against simplifying such motivations just to this aspect, where “gender, culture and class and individual dispositions” (p.703) play an important role in the complex make-up of a nursing professional. These views may also change as students progress in their courses. A longitudinal study of nursing students in The Netherlands found that even though many students entered their courses with altruistic and empathic predispositions, their perceptions towards nursing changed to being more professional and focused on their role, knowledge and skills [6]. Conversely, this was not reflected in the current study where nurses still displayed strong altruistic characteristics beyond graduation from their nursing courses. This suggests that the caring aspect of nursing and ability to make a difference to people’s lives should be emphasised in recruitment to the profession. Additional research in the Indonesian context is needed to better understand the influences on nurses’ personal dispositions and whether these change over time.

Nurses and nursing students in this study both described a personal calling into nursing. Calling, itself, has been described as complex in nursing, and having changed from a traditional perspective based in religion and femininity, to a more contemporary conceptualisation focused on care provision, the profession and self-fulfilment [19]. In this study, a more traditional focus emerged with both focus on serving community and service to God. This may be, in part, related to the fact that the study was undertaken in a faith-based university and health care group. Being a strong faith-based country, this may be a particularly important consideration in Indonesia and would benefit from further research with other groups across the country. The importance of inclusion of this concept in recruitment into nursing in the country could be further explored. In a recent study in Indonesia, the concept of calling and reason for entering nursing played a role in student success in a nursing program [20]. The importance of understanding values is particularly pertinent in nursing recruitment strategies with a recent mandate in the United Kingdom for values-based recruitment of healthcare students aligning with those of the National Health Service [21].

The influence of families and others was a factor in this study for pursuing a nursing career. In Indonesia, families have been shown to play an important role in career pathways, particularly in family businesses [22]. However, the literature is mixed on whether this is an important factor for nursing. In their review, Wu et al. [9] identified that some studies identified parents as not being supportive of their children entering the nursing profession because of low pay and status, a view reflected elsewhere [23]. Despite this, families have been found to be a strong influencing factor influencing choice of nursing career in some studies [8, 24, 25]. Having family members or friends who are nurses or had experienced time in hospitals were identified as influencing factors in one study [8]. In a study conducted in the United States, Woods-Giscombe et al. [25] recommended including family members into recruitment processes into nursing, particularly for recruitment of students from underrepresented groups. This suggests that recruitment strategies should not only be directed towards potential students, but their families as well.

While career stability and vocational reasons have been identified by other researchers as guiding factors in pursuing nursing careers [8, 18], these aspects were not identified by nurses and nursing students in this study. It is possible that cultural aspects may play a role. In a Norwegian study, nursing students from immigrant backgrounds were found to be more motivated by salary, status, and work flexibility than non-immigrants [26]. Findings from the current study suggest that such considerations might not be primary considerations for Indonesian nurses and students and that more research is needed to explore this aspect further.

Media representations have been identified in a number of studies as influencing decisions to pursue nursing careers. In one Australian study, hospital dramas on television as well as print and television news played a role in influencing graduate entry students to pursue nursing education [8]. In another Australian study focused on television representation of the nursing profession, nursing students perceived nurses to be negatively represented in comparison to doctors who were positively portrayed. They recognised that medical programs could provide some recruitment value [27]. However, a role of media influencing career choice was not identified in this study. Whether or not this plays some role in assisting career decisions for Indonesian students could also be examined further.

There are some acknowledged limitations to this study. The sample was drawn from one faith-based university and hospital group. While the study population was large and drawn from a number of locations, findings may be different in other Indonesian nurse populations across the diverse cultural groups in the country. Furthermore, data were only collected using one open-ended survey question. Further research that explores these concepts in greater depth would be highly valuable.

## Conclusion

With global nursing shortages, there is an ongoing need for effective recruitment strategies into the profession. This makes it vital to understand motivations of those entering the profession to facilitate recruitment approaches. However, motivations may vary according to a wide range of intrinsic and extrinsic factors. This study identified that Indonesian nurses and nursing students were largely motivated by a need to serve others and God, personal calling, and the influence of family. As a strong faith-based country, this is likely to be an important consideration in future nursing recruitment. However, further research is needed across more communities to ensure that other motivating factors can be identified and incorporated into successful recruitment strategies. Further research is also needed to understand if these concepts play a role in nursing students successfully completing a program and entering the nursing profession.

## Data Availability

The datasets generated and/or analysed during the current study are not publicly available due to ethical approval conditions but are available from the corresponding author on reasonable request.
